# Supplementary figures and images for: Intra-host changes in Kaposi sarcoma-associated herpesvirus genomes in Ugandan adults with Kaposi sarcoma
Source: PLoS Pathog. 2021 Jan 19;17(1):e1008594. doi: 10.1371/journal.ppat.1008594 (PMC7845968; doi:10.1371/journal.ppat.1008594)

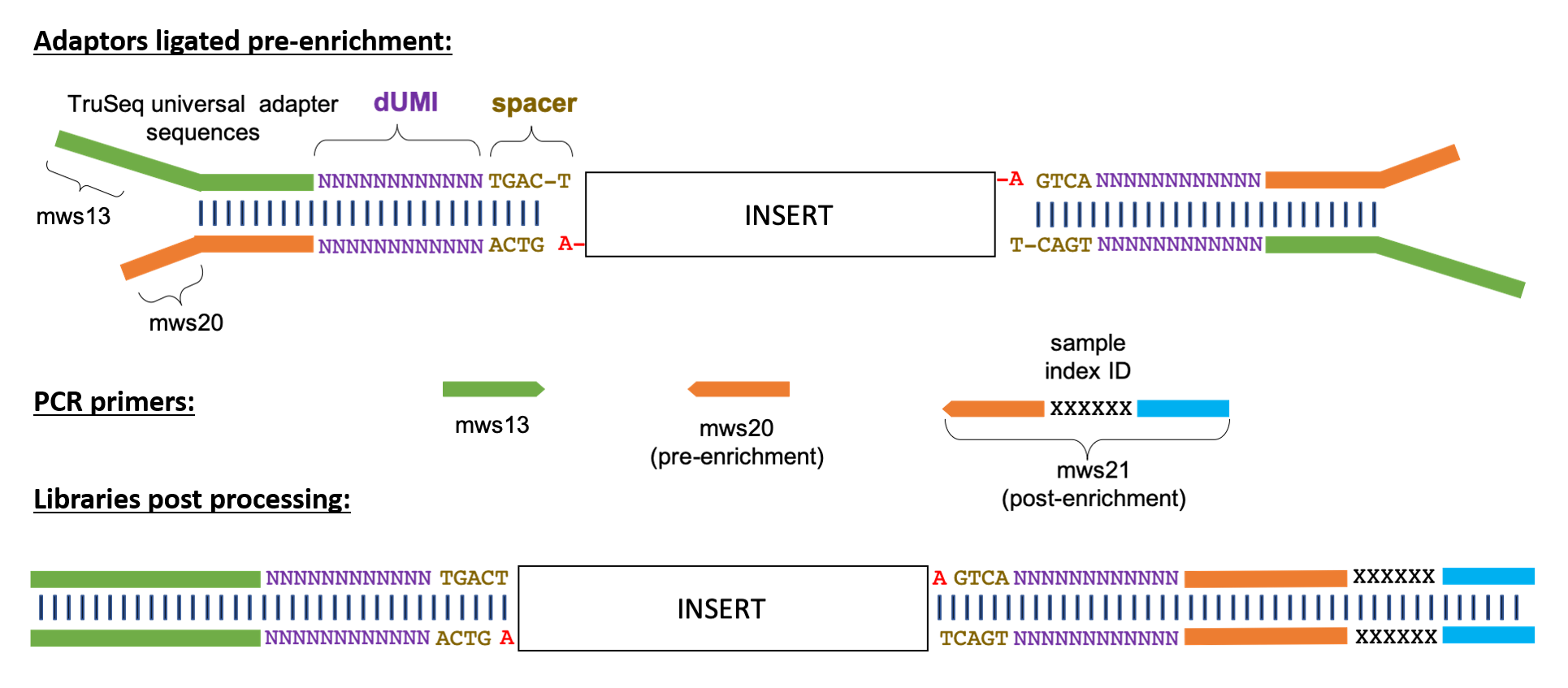

Supplement: S1 Fig — During library preparation, sheared DNA fragments were A-tailed and ligated with forked, double-stranded oligonucleotides containing Illumina TruSeq universal adaptor sequences, 12-random base pairs as dUMI and spacer sequences. The adapted DNA libraries were PCR amplified before enrichment with primers mws13 and mws20, which bind to Illumina Truseq adaptors. Primer mws21 containing sample index ID for multiplex sequencing was used for PCR following enrichment. DNA libraries post processing are shown at the bottom. (TIF) [file ppat.1008594.s001.tif]

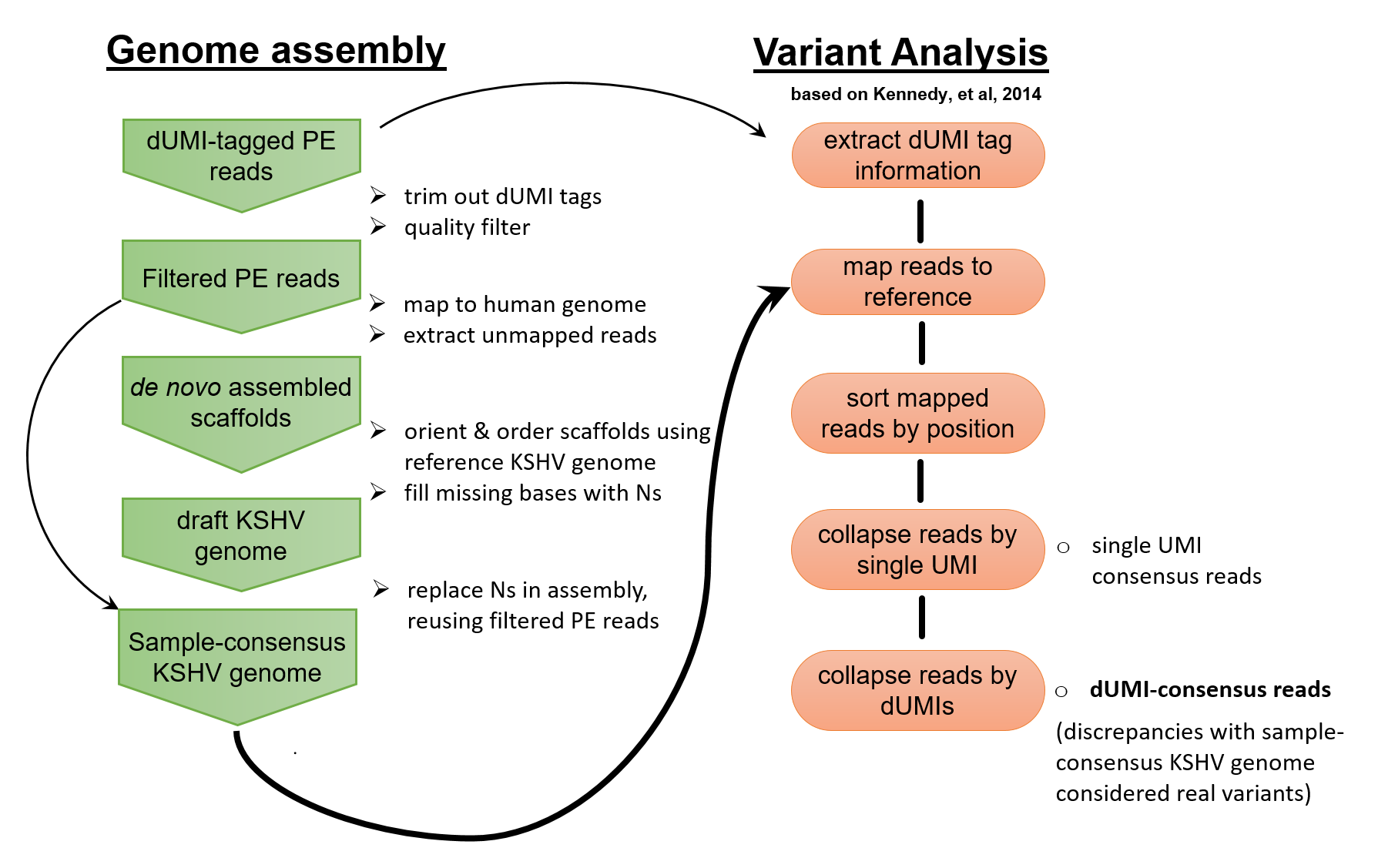

Supplement: S2 Fig — KSHV genomes were first assembled de novo from sequence reads of each sample, before being used as reference for mapping their respective dUMI-consensus reads (adapted from [61], see details in the Methods section). Discrepancies in bases between the sample-consensus genome and mapped dUMI-consensus reads were taken to be real intra-sample variants. (TIF) [file ppat.1008594.s002.tif]

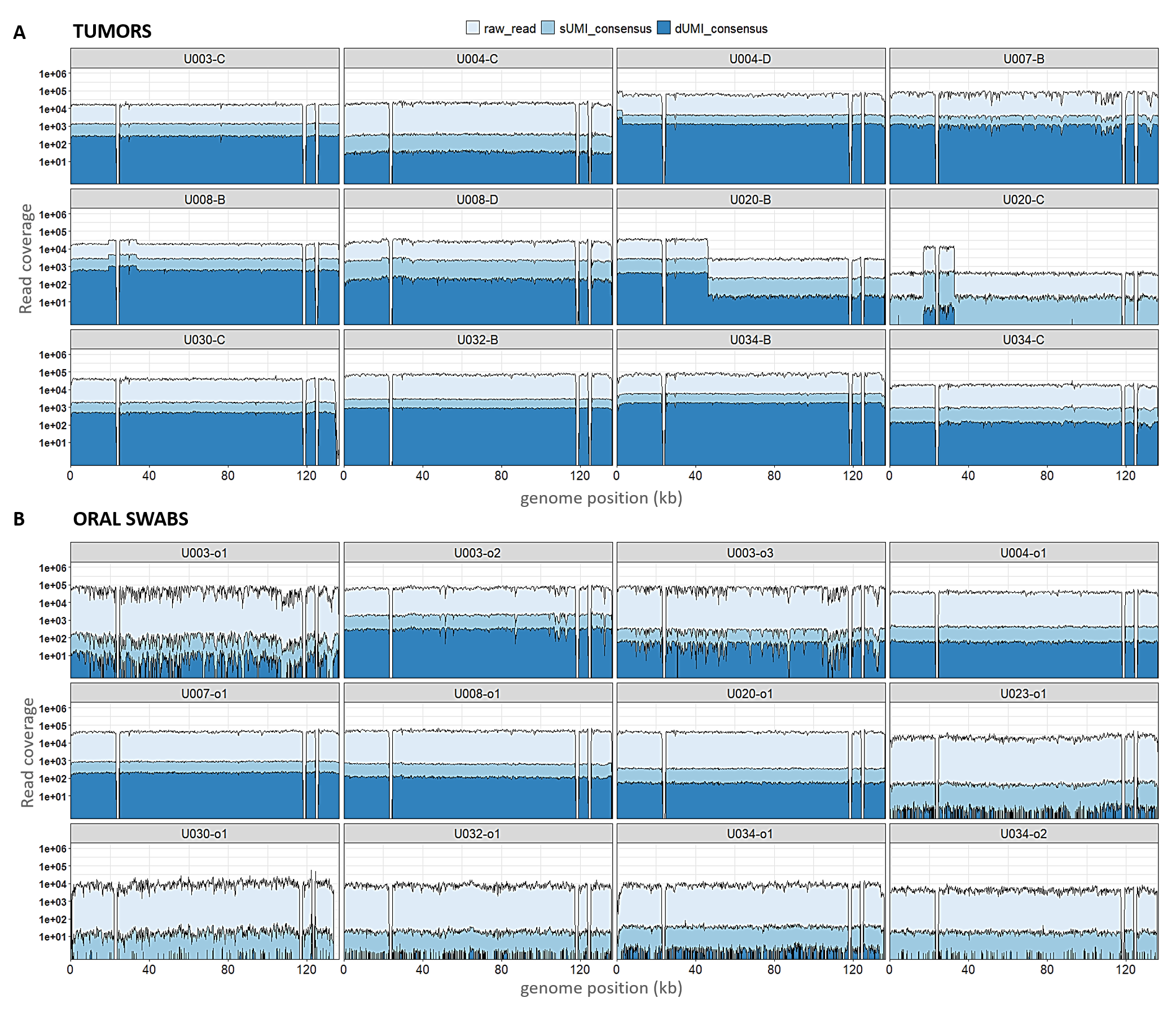

Supplement: S3 Fig — Raw (light blue), sUMI (blue) and dUMI-consensus (dark blue) read coverage in log scale along the de novo assembled, sample-consensus KSHV genomes in tumors (A) and oral swabs (B) examined in this study. Major repeat regions were masked and seen here as no coverage regions. (TIF) [file ppat.1008594.s003.tif]

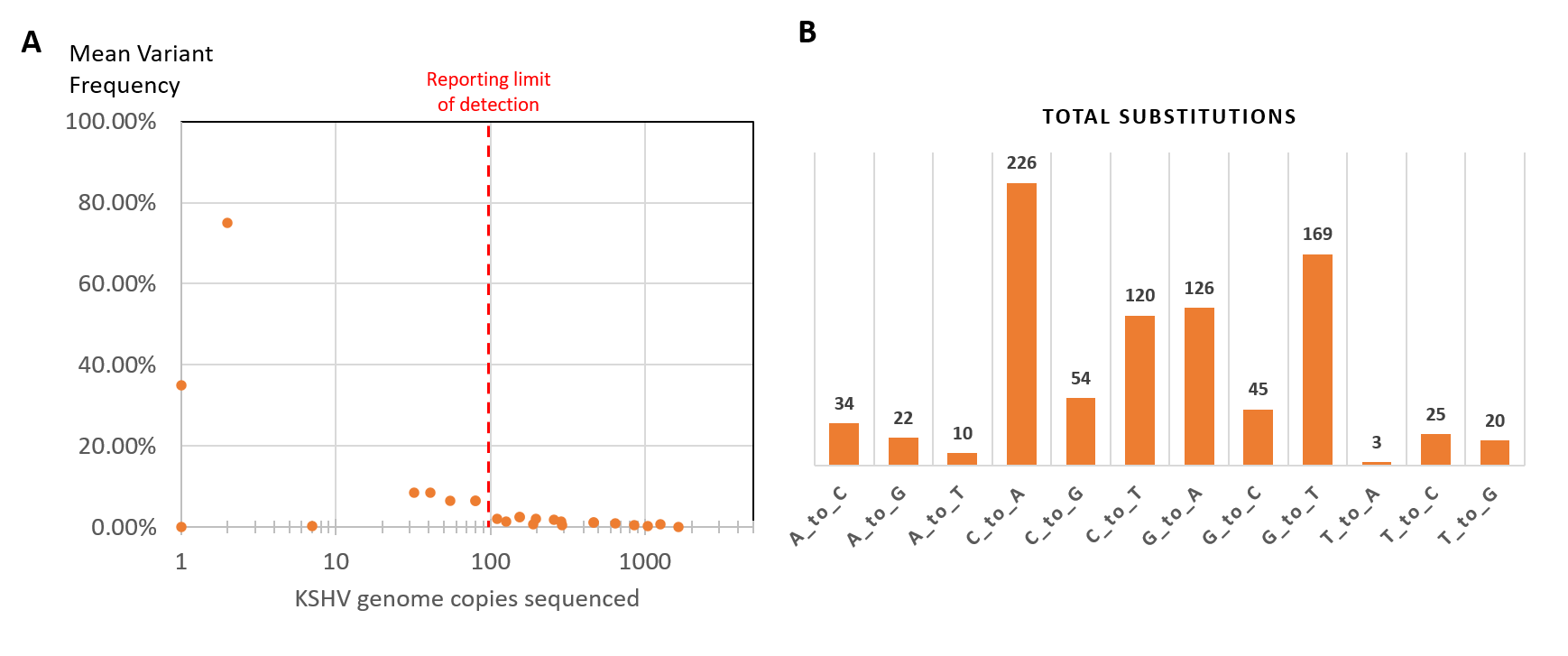

Supplement: S4 Fig — (A) KSHV intrasample variant frequency as a function of read coverage. Sample variant frequencies were shown in Table 1 when at least 100 viral genomes were sampled, since below that level, minor variant frequencies were judged to be unreliable. (B) Intra-sample minor variants detected in dUMI-consensus reads of all samples by type of base substitution. (TIF) [file ppat.1008594.s004.tif]

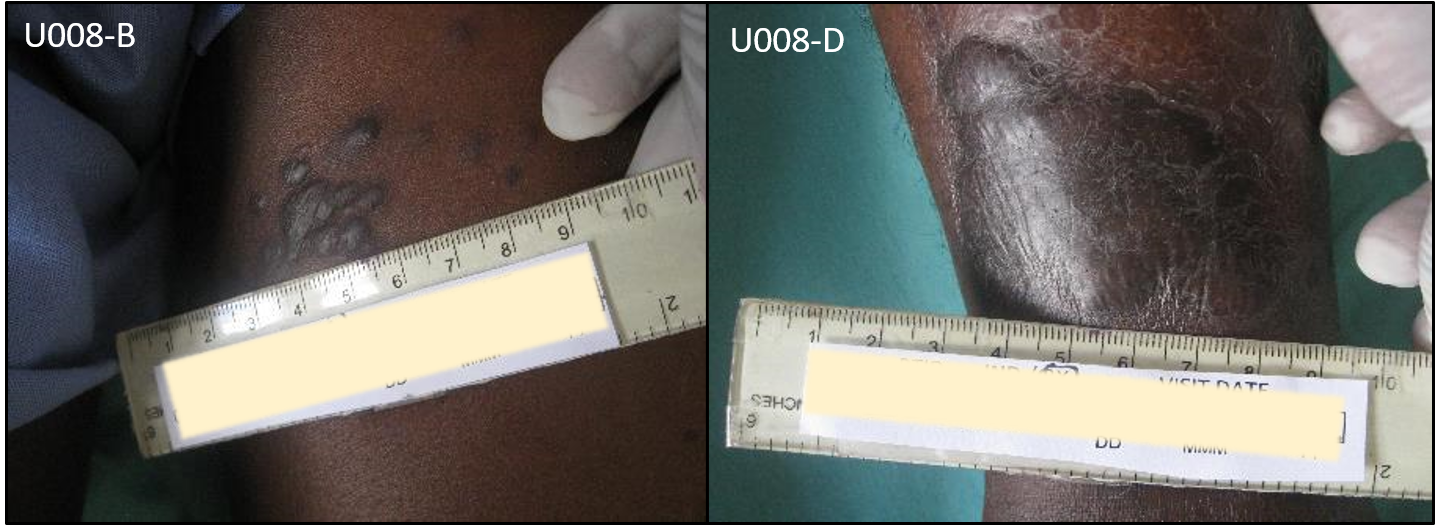

Supplement: S5 Fig — U008-B biopsy was obtained from lesions in the upper thigh, while U008-D was biopsied from a large lesion on the knee. (TIF) [file ppat.1008594.s005.tif]

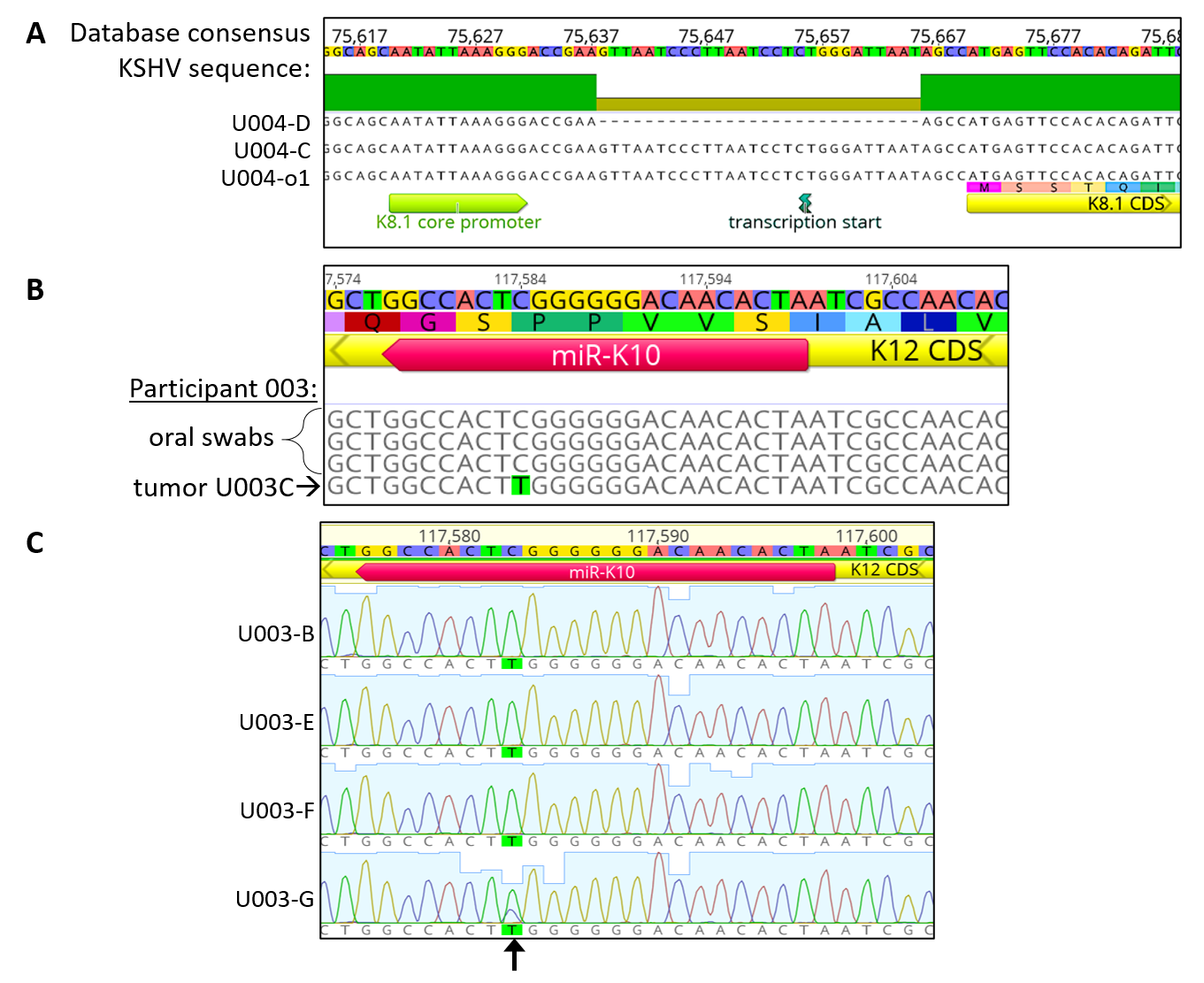

Supplement: S6 Fig — (A) Alignment of KSHV genomes from Participant 004, showing a 28-bp deletion in the K8.1 promoter in U004-D. U004-D and U004-C are from tumors while U004-o1 is from an oral swab. (B) The only intra-host synonymous mutation found in this study, within miR-K10 in participant 003. (C) Sequence chromatograms of miR-K10 in other tumors of participant U003, with a T in all tumors and a mixture of T and the database consensus C in a minority of viruses in U003-G. (TIF) [file ppat.1008594.s006.tif]

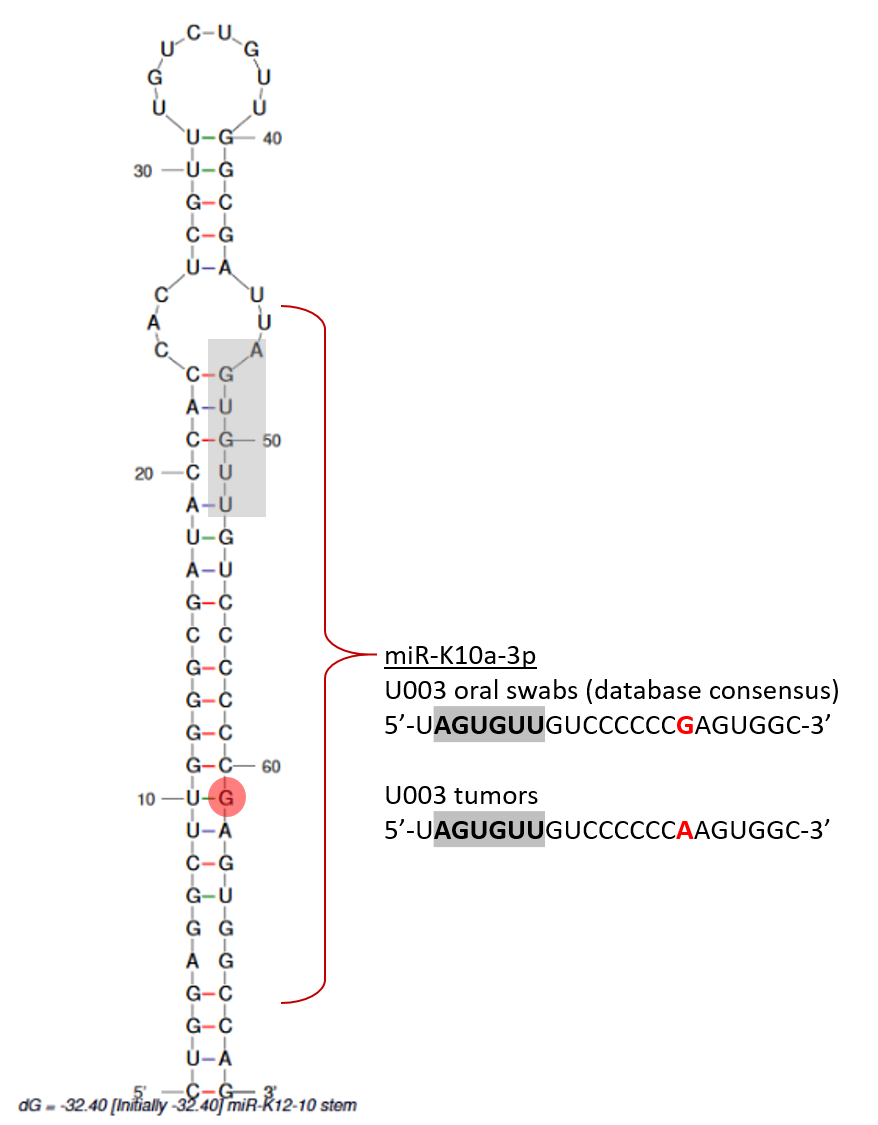

Supplement: S7 Fig — The structure of pre-miR-K12-10a was predicted using mfold (http://unafold.rna.albany.edu/?q=mfold/RNA-Folding-Form), indicating the mature miRNA, seed sequence (grey) and the intra-host polymorphism (red) found in participant U003. The G➔A change in RNA sequence resulted in a slightly more stable stem loop (ΔG -32.40 ➔ -32.70). (TIF) [file ppat.1008594.s007.tif]
