# Supplementary material for: Intra-host changes in Kaposi sarcoma-associated herpesvirus genomes in Ugandan adults with Kaposi sarcoma
Source: PLoS Pathog. 2021 Jan 19;17(1):e1008594. doi: 10.1371/journal.ppat.1008594 (PMC7845968; doi:10.1371/journal.ppat.1008594)
Supplement: Attachment — Submitted filename: Reviewer responses_final.docx [file ppat.1008594.s012.docx]

Reviewer's Responses to Questions

**Reviewer # 1**

Major comments:

*1. The study could benefit enormously from including an expert in KSHV and herpesviruses. For instance, the premise that different subtypes of KSHV are associated with different disease manifestations is far from certain (line 79). The EBV data cited in the introduction, likewise, are still widely debated. The field has been down to many false paths due to overblown claims of that nature. The authors say as much on line 88.*

Response: We agree that KSHV subtype association to disease manifestations is far from certain. The sentence following former line 79 noted by the reviewer attempts to highlight this uncertainty: "However, correlations of KSHV genetic subtypes with virulence has not been consistently observed." We have combined these sentences and omitted extraneous information to better convey this uncertainty.

Regarding the suggestion for the study to include KSHV experts, co-authors Drs. Casper and Phipps led the parent project and are both experienced herpesvirus researchers. Dr. Casper has published 49 papers on herpesviruses, including 32 on KSHV over the past 18 years. Dr. Phipps has published 9 papers on KS or KSHV and 2 on other herpesviruses. Dr. Michael Lagunoff, an active member of Mr. Santiago’s PhD thesis committee and author of 33 papers on KSHV, has also reviewed the revised manuscript.

*2. Likewise, it is not at all clear if genome recombination is a consistent phenotype of KSHV or an artifact of lab culture. Recombination is rare and not normally associated with disease scores in any of the herpesviruses.*

Response: We also agree that recombination is rare and has not been associated with different disease manifestations. In our original version of the manuscript, we referred to KSHV recombination as having occurred in the distant past resulting in mosaic genomes. To make our point clearer, added "ancient" and "resulting in mosaic genomes" to this sentence (line 88). We further comment on the rarity of KSHV recombination in the Discussion, new lines 484-486.

*3. The authors overstate the significance of unique molecular identified for DNA sequencing and variation discovery of DNA material. The myriad of FDA-approved tests in cancer as well as other studies show that in the case of DNA, sufficient read depth can overcome many of the concerns raised in the introduction. Errors due to NGS methods play a much larger role in sequencing RNA viruses, as reverse transcriptase has a much higher error rate than modern DNA polymerase used for NGS.*

Response: The reviewer may be referring to cancer tests that interrogate clonal mutations (i.e., mutations shared by a substantial proportion of genomes within a population). However, these approaches are fundamentally limited in their ability to identify rare variants in heterogeneous mixtures, regardless of read depth (reference: Salk, Schmitt, Loeb. Nat Rev Genet 2018). Furthermore, given that herpesviruses have a lower error rate than RNA viruses, NGS errors correspond to a GREATER (not lower) proportion of suspected mutations for herpesviruses. The use of duplex UMI, which reduces the error rate approximately 5 orders of magnitude below that of typical NGS, is therefore essential for accurate low-level variant discovery and confirmation, which was our goal. That duplex sequencing is greatly superior in removing sequencing errors and therefore rare variant identification is illustrated by the read reduction data shown in Figs 1B and S3, which compares raw reads (as the reviewer advocated) to single and duplex UMI-consensus read coverage. This clearly shows the value of error rate reduction to arriving closer to describing the actual viral population.

We added lines 468-471 in response to this critique.

*4. It would have been better if in a manuscript about KSHV more relevant references about KSHV were provided and less for EBV and HIV. The dUMI technique and related approaches are sufficiently developed and now commercially available. There is no need to go into extensive detail about it.*

Response: We included references on KSHV which we believe are important for explaining the rationale for our study. Importantly, the literature on KSHV viral strains in *in vivo* KS samples is quite limited, which was a key motivation for us to conduct this study. Because EBV is the other known gamma-herpesvirus and has been much more extensively studied than KSHV, we included discussion of EBV as a potential model for KSHV pathogenicity. HIV was discussed in the manuscript to indicate that all our study participants were HIV co-infected.

Since this was the first time dUMI sequencing technology had been used in this field, we feel that it is necessary to briefly describe it in order to explain to readers how its capabilities address the shortcomings of previous studies on KSHV diversity. Indeed, this reviewer indicated in a later point that the use of “dUMI is novel and truly instills confidence in the analysis“. Reviewer 2 noted that “The novelty of the manuscript lies in the application of the high-depth dUMI method and the resulting high-accuracy base calls and description of intra-host variation that has previously only focused on tumor samples from the same subject and never been reported at this depth.”

As with our preceding response, new lines 468-471 addresses the latter part of this critique.

*5. Sequences need to be submitted to GenBank before acceptance.*

Response: GenBank accession numbers are now available and have been added to the manuscript in the Methods, as well as appended to Table 1.

*6. The use of dUMI is novel and truly instills confidence in the analysis. It suggests that in the control ~ 5 reads give rise to one dUMI and above further collapse 50 raw reads per dUMI (line 251). How do the authors explain the difference in tumor vs oral dUMI coverage? The tumor samples all seem to work very well, but the majority of oral samples failed to yield satisfactory dUMI coverage, even though raw read coverage was similar to tumor coverage?*

Response: dUMI-read coverage depends on the initial number of DNA templates present and their quality. Oral swab samples had less virus, and as well, DNA from ~75% of oral swab samples were too degraded for use. We therefore enriched oral swab libraries twice and PCR-amplified with more cycles to achieve minimum DNA amounts for quality control before Illumina sequencing. Thus, oral swab libraries had a higher proportion of PCR duplicates. We added text to the revised manuscript explaining this information, current lines 291 - 296.

*7. There is an alternative explanation to breakpoints not supported by junction reads, at least theoretical. Any KS tumor includes full-length latent genomes, perhaps defective genomes, perhaps integrated genomes, but also encapsulated virion DNA and, defected, replication intermediates. The proportion of these varies depending on the number of lytic cells in the lesions.*

Response: Referring to previous work on EBV, we noted in the original manuscript that despite a search, we found no significant support for host cell genome integration. To emphasize this, we added lines 504-505 to the Discussion: "All breakpoints detected in our KSHV sequence reads, when mapped, connected to other regions of the KSHV genome."

Second, as was originally noted, aberrant genomes we identified were the dominant KSHV genome sequences in each sample in which they were observed. In response also to Reviewer #3 summary/comment 3, lines 500 – 503 were added.

*8. It is in the discussion where this paper veers off course. The authors did not generate ">100 error-free genomes" line 444, but only 12 for 12 tumors. The majority of oral samples have less than sufficient mean coverage.*

Response: We intended to indicate that over 100 distinct genome copies were sequenced per tumor from most tumor samples. (And these all had virtually the same sequence, given the very little intra-sample diversity.) Creating a consensus of all KSHV sequences within each tumor sample, we subsequently obtained 12 sample-consensus genomes from 12 tumors. KSHV genomes were distinguishable only when comparing across individuals. We revised the statement to as follows: "KSHV genome sequences were obtained essentially error-free from 11 oral swabs and 12 KS tumor samples provided by 9 Ugandan adults with KS. More than 100 genome copies per sample were sequenced from most tumors, while fewer copies were sequenced from oral swabs due to lower input and sample quality." (from current line 462)

We can be certain of the numbers of KSHV DNA molecules sequenced per sample because each dUMI tag consensus is derived originally from 1 DNA template molecule, which allows us to count the number of template molecules sequenced in the end. As noted in original lines 253 – 255 (current lines 265-267), "Since each dUMI tags a unique DNA molecule before PCR, the number of unique dUMI tags indicates the number of unique viral templates sequenced." (please refer also Table 1 and response to question 4 above).

*9. The conclusion that the observed intra-tumor rearrangements were not random (line 465) is premature. First, the limited number of samples does not allow for a statistically significant answer to this hypothesis. Second, during latency any mutation outside the genes required for latent persistence is neutral. While mutations that interfere with latent replication are selected against at every cell division. It is premature to speculate on the oncogenic benefit of any of the observed rearrangements (line 470) or specific selection pressures against K8.1. (line 509).*

Response: We agree that cannot conclude that any of the genomic aberrations observed were selected for or biologically significant based on this study alone. That said, we observed some striking patterns in the mutations that we felt would be worth hypothesizing about how they may have arisen and potential biologic relevance that could be pursued in future studies. We have revised most of the Abstract and Discussion to be clearer that the discussion of possible biologic significance of the observed mutations is intended to serve as hypothesis generation for future studies.

Minor comments:

*1-2.	On line 253, the authors reference Figure 2B, which does not exist.*

*3.	The author’s reference a previously described duplication in BAC-36 in line 261, and have incorrectly reported the duplication size as 19kb, instead of 9kb.*

Response: We thank the reviewer for these. They have been corrected.

*4. Table 1, page 13. The table is not formatted properly and indicates a missing field on the left-hand side. The mean read coverage seems to represent the number of reads and maybe misleading when looked at with supplementary Figure 3, which reports read coverage. The authors don’t describe if data was normalized. Please use the median instead of mean coverage and provide 95%CI or sd in table 1.*

Response: We thank the reviewer for suggesting more informative statistic measures and have updated the tables and text (original lines 280-282; current lines 289-291). Tables are embedded in the draft manuscript following the instructions of the publisher, and Table 1 starts with Pt ID as its first column. The median read coverages are concordant with the plots in Figure S3. The read counts we report are absolute as we are not certain how best to normalize them.

*5. The Authors describe the inversion of the K8.1 exon in Tumor U003C, Line 330. The legend in Figure 5, describes the presence of the faint band seen in the BCBL-1 control TR-K8.1, Fig. 5D, as the presence of the forward primer amplicon. Perhaps different primers can be designed to confirm the band/or lack of a band in the control sample. It does not clearly state that the bands found in the control sample were confirmed by Sanger sequencing to be the forward primer amplicons. The text describing the data in Figure 5C in line 335 is different from the figure legend. The Figure and its legend indicate that the samples being compared are from different individuals as opposed to the same patient, as described in the text. There is a Typo in line 330, A ‘U’ is missing in the tumor sample number.*

Response: We thank the reviewer for noting this error. We have clarified Fig 5D legend to mention that the light bands from PCR of K8.1-TR junction were assessed by Sanger sequencing and found to result from weak binding of the forward primer. We updated line 335 (current line 351) to indicate, "from an unrelated tumor (**Fig 5C**) and BCBL-1 (not shown)" and corrected the typo in the sub-section header. Elsewhere we had mentioned (expanding to current lines 390-392) that we could not find a functional forward primer binding inside TR after 3 attempts.

*6. Line 368, the authors describe the breakpoints inside ORF11 and ORF18 and fusion into the TR. PCR primers are designed and confirm the 3’ fusion of ORF18 and the TR but not the fusion of ORF11 and the TR at the 5’ end, Fig. 7F, row 4. This is also shown in Fig. 7G, lane 4.*

Response: As stated from original line 374, we attempted to design other forward primers binding within TR sequences for Fig 7F - 7G lane 4, but none produced PCR products on a BCBL1-DNA template, pairing with the reverse internal TR primer that is known to work (based on Fig 7G lane 5). Since this question was raised by all reviewers, we have further expanded on this in the text, starting on line 391 of the revised manuscript (as well as adding a new reference [82)).

*The authors did not explain the presence of faint bands in Fig 7G, lane 7, 8, and 9, which may indicate the presence of these PCR products.*

Response: We thank the reviewer for this observation. We had only briefly addressed the light bands in the original Fig 7G caption ("…Attempts to sequence the light bands in U020-C #7 and #9 were unsuccessful…"). Where sufficient DNA could be extracted from the light bands, we had only found irrelevant sequences. We now add more details about these attempts and on all other bands to the Fig 7G legend.

*Figure 7F, lane 5 referenced in line 377 is the same PCR product as the 3’ junction product reported in line 374, Figure 7G, lane 5.*

Response: We had mentioned Fig 7F, lane 5 to illustrate that the reverse internal TR primer we used elsewhere is functional; the product in lane 5 had no relation to what is expected for lane 4. Additions to current line 391 onwards were added to help make this clearer.

*The wild type product of lane 2 in Figure 7F and G is much larger than the expected band size. In conclusion, no PCR confirmation was provided to verify this finding.*

Response: We thank the reviewer for bringing this error to our attention. The expected size of the amplicons in lane 2 in Fig 7F and 7G were 969 bp (Supp. Table S1B). As we note in summary comment 4 of Reviewer #3, the expected sizes were estimated on the published reference genome of isolate GK18. Ultimately, this PCR product was confirmed to be the breakpoint junction by Sanger sequencing. We updated the Figure 7 table to reflect the correct band sizes.

*7. It could be argued that mutations are called when a low number of reads obtained, despite selecting mutations with a frequency of 100. As mentioned in their references, these mutations could be a consequence of DNA damage and other cellular events.*

Response: As explained in Methods, dUMI-consensus reads were mapped to the *de novo* assembled, sample-consensus genome sequence, and only "Discrepancies between mapped dUMI-consensus reads and the sample-consensus genomes… were considered to be sequence variants that existed prior to PCR amplification."

Samples with median dUMI-consensus read coverage below 100 did not pass our quality check and hence were not analyzed by dUMI, even if they had enough raw reads for assembling full-length genomes. dUMI consensus reads have been reported to avoid artifacts from DNA damage and can result in accurate sequencing of single DNA molecules regardless of lower numbers of consensus reads or DNA damage, since it is exceedingly unlikely for complementary artifacts to occur on both DNA strands of a single molecule at the same position (reference: Schmitt et al PNAS 2012).

We modified current lines 319 and 475-477 to make more explicit that we cannot fully exclude the possibility of artifacts.

*8. The resolution of the Figures could be improved on.*

Response: We have sharpened the figures and made them slightly larger.

*9. (line 301) If variants were supported by only one read, where these excluded from the consensus sequences submitted to GenBank?*

Response: Yes, they are not part of the submission since there can be dozens of such very low-frequency variant base in a sample (Fig 3A), and their significance is hard to interpret, being within the published resolution of the assay.

*10. line 417: Did this SNV disrupt the target sit of miR-K12-10? Please provide a mfold structure comparison.*

Response: No, it did not. We thus added this information and mfold structure prediction in the Results (lines 438-439), Discussion (lines 491-493) and Supplementary Figure S7.

*11. line 439: Would this technique detect HIV, since it does not use reverse transcriptase or was HIV assayed by viral load assay?*

Response: This search would only be expected to find integrated HIV proviral DNA if detectable. The use of a KSHV-enriched library, as well as the fact that HIV-infected cells are known to be quite rare, makes the likelihood of detecting HIV sequences vanishingly low. We omitted reference to HIV in this sentence.

*12. Please remove line 521 "or with a helper virus" as there is no evidence for reinfection.*

Response: We agree that reinfection with a second virus is unlikely, but it is possible that an intact version of the primary infecting KSHV virus remains in the tumor cell along with the defective virus, albeit at very low levels in some cases. Metastasis is just one possible explanation, as we cannot yet a possible contribution of helper viruses carrying defective genomes to other sites. In all instances of genomic deletions, the terminal repeat sequences, which contain the putative packaging (pac1) and cleavage sites, were still present. Reviewer #2 also suggested that expression from intact genomes might complement the propagation of the defective genomes. We added discussion on this in current lines 560-573.

**Reviewer # 2**

*1. The findings of genomic aberrrations in tumor-derived but not in the oral samples are quite interesting but the results are perhaps over-interpreted as being tumor drivers (lines 51-52 and elsewhere) as opposed to a consequence of the genomic instability inherent in tumor microenvironments that may adversely impact the stability of KSHV episomes in the course of their replication and partitioning to daughter cells.*

Response: We agree with the reviewer that the genome instability inherent in tumors might contribute to the observed KSHV genomic aberrations. Since we cannot evaluate these alternative hypotheses rigorously with our results, we made more explicit throughout the Discussion that the significance of the rearranged KSHV genome is unclear (e.g. current lines 506-507). We included discussion of the possible mechanisms by which the IR1 regions or K8.1 gene could be selected for or serve as tumor drivers as hypothesis to explore in future studies but not as conclusions that can be drawn from our current data (current lines 541-542). We have attempted to make this intent clearer throughout the revised manuscript.

*2. Moreover, the finding that a signature aberration was found in more than one tumor is strong evidence for metastasis of a KS tumor, in the likely absenece of the capacity for the etiological agent to spread via an extracellualr virological mechanism. This reviewer felt this was an important finding that was curiously not highlighted to any significant degree in the manuscript.*

Response: We agree that this is an important finding that should be highlighted more in the manuscript. In response to this common concern of Reviewers 2 and 3, we expanded more on this finding in the Discussion (current lines 560-573), Abstract and Author summary.

*3. In light of previous findings, the justification for doing the work and for doing the work at the incredible level of sophistication was somewhat lacking given that the outcomes largely showed what might have been anticipated from the literature, with the exception of the genomic duplications, inversions and deletions. But as stated above, the significance of these in pathogenesis remains debatable.*

Response: One of the goals of the study was to determine if observations regarding lack of intra-host KSHV diversity observed in some (but not all) previous studies could be more definitely defined through the use of new sequencing approaches. In our study, we believe that we have fairly conclusively shown that intra-host KSHV “quasispecies” or diversity within the viral population is negligible, confirming what has been described in previous work. However, as pointed out by reviewer #3, “This is in contrast to some earlier studies with analysis of specific KSHV genes, showing KSHV sequence variability and tissue compartmentalization, and will be important to document such findings”. Indeed, originally in the Introduction (lines 89 – 94) and Discussion (original lines 453-455 /current lines 479-483), we cite the few studies reporting some KSHV intra-host diversity, and additionally noted that these studies were largely derived from PCR of a few genes – ours was the first study to examine this issue with complete genomes at depth. Second, the diversity we observed was in fact lower than had been appreciated previously, and this was only made possible by the technologies we employed (see response 1 to Reviewer #1). Third, “the genomic duplications, inversions and deletions” were novel and their potential significance was discussed. Lastly, we agree that the significance of these observations to tumor pathogenesis is not understood. We made more explicit our ambivalence to their possible significance throughout the Discussion, as well as revised the Abstract and Author summary.

*4. There was some confusion in the presentation of the number of samples being analyzed versus those actually being being compared tissue to tissue. See Line 31 versus 107-108*

Response: We appreciate the reviewer highlighting the confusing descriptions. We have modified the Abstract text (line 24 and 30). Details of the matching pairs of samples are explained in the Results.

*5. In the PCR experiments designed to interrogate the genomic aberrations and identify the breakpoints by Sanger sequencing there are instances where one side of the junction is clearly identified and the other is only evident from the deep sequencing method. While this reviewers interpretation of the findings is largely in agreement with the authors', there is not real explanation offered as to why one junction is readily detect and another is not since they should be equimolar in the sample amplification reactions.*

Response: This issue was addressed in original lines 374-377 ("… the latter result is likely due to GC-rich TR sequences being largely unsuitable for primer binding"), but since all reviewers had confusion on this part, we expanded on this to make it clearer (new lines 390-392, including new reference [82]).

Minor comments:

*1. Some discussion of why a secondary library enrichment of low copy# samples does not bias the sequencing outcomes.*

Response: We see no evidence of that the sequencing outcomes were biased, as mentioned in the Results section (current lines 340-344). To further clarify, we added more in the Discussion on this, to the original line 456/current line 482-484: "While second enrichment could conceivably bias sequencing results, comparing doubly-enriched oral swabs libraries to their matching, singly-enriched tumor libraries show identical KSHV sequences."

2. LINE 247- POLG not defined

Response: We added its definition, DNA polymerase subunit gamma

3. LINE 275- How does going from a median of 0.17% to 1.3% equal a 6000-fold increase?

Response: We thank the reviewer for noting this error. We have now omitted this statement and report only the final enrichment numbers to both make the table simpler and allow room for the additional information requested.

4. LINE 466-469- Brings up a good concept but sufferer from the lack of comparison to data from immunological studies to suggest the immune response to the gene products indicated are associated with protection against KSHV disease. The authors should discuss since Whitby and others have conducted whole proteome B and T cell repertoire screening without defining patterns of immunodominance or correlates of protection.

Response: To clarify, we had not made an assumption that immune response to any viral protein products could be protective against pathogenesis of KS, but merely proposed that immune response might influence continued proliferation of already established tumors that express viral proteins. Tumor cells that have lost expression of targeted viral proteins may be selected for, especially if the targets were unnecessary for continued growth. We also thank the reviewer for bringing attention to those studies. While these studies have shown that most KSHV proteins can serve as T-cell epitopes, they also show that the ones that elicit the strongest responses differ greatly between individuals. Characterizing T-cell epitopes of the individuals in our cohort will be needed to corroborate any appearance of selection against a particular KSHV gene. We thought it best to replace the statement ("The mutations may have been selected for, if for instance expressing these proteins exposed host cells to immune targeting.") with clarifications in lines 521 and 556-558.

5. LINE 480- RNASeq or transcriptomic/array data, in this reviewer's experience, seems to suggest that there is no single pattern of KSHV expression associated with tumors. Almost exclusively lytic exclusviely latent, and mixed patterns have been detected in association with similarly staged tumorigenesis, so I not clear as to the accuracy of the statement.

Response: While we are aware of the studies showing the bimodal viral transcriptome profiles of KS tumors in vivo, it seems that there were detected lytic transcripts in all cases. The reference we cited only suggested the importance of residual lytic transcription to persistence. The other reference alludes to that inhibiting herpesvirus lytic replication processes by therapeutic means have been known or used to arrest KS proliferation and progression. We did not change this statement.

6. LINE 489-491-This seems like a list of genes and functions without a clear linkage to the discussion above it. Is the suggestion from the RNASeq data from the same group demonstrating up or down regulation of these genes in concert with the IR1 aberration? The conclusion is not evident and could be clarified.

Response: This paragraph is a discussion of the importance of IR1 and adjacent sequences to the KSHV infection cycle, in support of our hypothesis that some tumor-associated KSHV mutations can possibly aggravate or contribute to KS disease. Preceding this paragraph, we mention that the IR1 region of U008-B & D was duplicated next to a latency promoter, and were the only sections remaining in the deleted KSHV genomes of U020-B and C. We added, "all of which might enhance the survivability of a host cell..." (current line 539).

We do suggest that for U008-B, the regulation the duplicated IR1 region (14.8 kb) is linked to its new location downstream the latency promoter, as RNA Seq data for U008-B (reference 81) shows that this chimeric sequence is being transcribed from the latency promoter in large quantities.

7. LINE 932-933- first statement is inaccurate since not all subject provided both samples.

Response: We add a clarification to the Fig 2 caption: "Each study participant contributed multiple KS tumors and oral swabs, but only those samples with the highest viral loads were reported here."

**Reviewer #3 summary:**

*1. Further discussion with emphasis on whether these mutations in KSHV caused tumorigenesis, or whether the rapid replication of cancer cells caused mutations in KSHV genomes, will be helpful.*

*The authors should consider moderating the implications of the genomic variation or genomic aberration of viral pathogenesis since it is not clear that either variation or aberration is driving disease, as opposed to a consequence of it.*

Response: We modified discussion of the tumor-associated KSHV mutations as being possible tumor drivers, and only as hypothesis to be explored in future studies rather than conclusions that can be drawn from our current data. Please see response to major comment #9 of Reviewer 1 and major comment # 1 of Reviewer 2.

*2. In lines 92 - 94 the authors mention that intertypic recombinants exist suggesting that co-infection of divergent strains must occur, but in fact it would seem to require co-infection of single cells with differential genetic subtypes. it is unclear whether the sequencing data presented provides evidence of such intracellualr KSHV diversity from a single cell to support the potential for forming intertypic recombinants. This comes up again in the Discussion Lines 458-459- is it co-infection of a single cell or leading to recombination, or accumulation of KSHV episomes leading to some sort of replication error by the cellular replication machinery trying perhaps to resolve the repeat structures in the KSHV episomes? It would seem that the probability of such, otherwise unlikely, events would increase with the episomal burden in cells.*

Response: Our study was not designed to determine intracellular KSHV diversity or the average episomal burden on each cell. And, since we sampled only 9 individuals, all within a relatively short time window, we made no claim on the frequency of co-infection or multistrain infection, except that it must be occurring sporadically based on recombination analysis by others (modified current lines 98 and 484-486). Please see response 1 to Reviewer # 1 also on recombination.

*3. Line 460-462- This reviewer agrees that the genomic aberrations are profoundly interesting and COULD be important causally, but it was unclear (perhaps my fault) what the frequency of such aberrations were of the total KSHV genomes from within a given tumor harboring those mutations. Is this frequency variable and does it associate with any other parameters of KSHV or HIV-1 co-infection- for example tumor morphotype.*

Response: The proportion of aberrant to wild-type genomes within tumors was variable. They are detailed for each individual tumor in the Results and mentioned in the Discussion (original lines 461-462). We now add to the Discussion lines 500-503: "Moreover, aberrant genomes comprised the majority of the KSHV genomes in the tumors in which they were detected, from 1.7-fold (U008-B) to 30-fold (U020-C) more aberrant than full-length intact KSHV genomes (Table 2 and Fig 7A-B). Further, no intact viral genomes were detected in 3 of 5 examined tumors from participant U003 (Fig 5 & 8)." (in response also to Reviewer #1 major comment 7)

With a small sample size, we cannot yet make any correlation to tumor morphotype or other clinical features of KS disease. We hope to address this question in a future study (current lines 581-584).

*4. Figures 6 and 7; Fig 6-Are the anticipated product sizes for amplicons (4 and 5) deemed to be diagnostic of the rearrangement and duplication correctly calculated because none seem close to those on the Agarose gels accept 1, 2, and 3.*

Response: Yes, these were deemed diagnostic, but there originally was uncertainty in the breakpoint coordinates, especially if at homologous repeat regions, and the expected sizes were estimated based on the GK18 reference genome. Ultimately, all PCR products were Sanger sequence confirmed. We have updated the table headings in Figs 6 & 7.

*5. Fig 7- The absence of seemingly critical confirmatory PCR products confounds the appreciation and absolute confirmation of the HTS data. For example Reaction 3 and 4, no product? So it is all based on the interpretation of reaction 5. The figure legend suggest the bands were excised and Sanger sequenced, but there don't appear to be diagnostic bands. So it would seem that one junction is a 'known and the other is implied from but incompletely define*

Response: We agree. The lane 3 result is most likely due to target DNA sequences being in the deleted region (1-3 reads/base). We revised from lines 392 onwards in the Results section on Tumor U020-C to indicate: "Finally, no PCR product was produced…"

Major comments:

1. Figure 5 C and D, it is not clear why 5D used BCBL and not U0007B as control, as in 5C?

Response: DNA from clinical specimens was extremely limited and irreplaceable, and we decided not to use them as controls in subsequent experiments. We did not revise the manuscript in response to this critique.

2. For U003 there are a number of protein altering changes in the saliva sequence as compared to the tumor sequences, where are these changes, how many of these changes are common and where do they differ, and how do they affect the proteins’ functions?

Response: We are not sure which part of the manuscript the reviewer is referring to. There were no more differences aside from those mentioned in the text, tables and supplementary tables, which is a testament to their remarkable conservation. Revised lines 482-484 and 488-490 reinforce this point.

3. Why did lane 4 of Fig 7G lane 4 did not show the 1kb PCR product if K2 is linked to TR as expected, while lane 5 did show a strong band that flanks the 3’ insertion site?

Response: This same concern was raised by the other 2 reviewers. We added more explanation for this result, starting from current line 391.

4. Fig 5S phylogenetic analysis is important since it compares the 9 KS sequences analyzed with the currently existing whole KSHV genome sequences, thus it should be shown as a regular figure, Moreover, the authors should comment on the UG156, why it is more closely related to the KSHV sequences from the western countries.

Response: We made the phylogenetic inferences into a regular figure (new Fig 4) as requested. We have no insight into UG156, which is not from our group but from Sallah, et al, 2018 (reference 36).

5. The KSHV sequences obtained are from homogenized tumor lysates, so how can the authors be certain that the final assembled KSHV sequence represent the actual viral genome in the tumor cells, whether multiple genotypes are within a single cell or in different tumor cell populations, or even are from extracellular viral particles.

Response: We cannot be certain of the cellular or extracellular origins. In any case, very few single nucleotide variants were detected (see Fig 3), which we suspect are further distributed to an average <1 non-consensus single nucleotide variant per individual KSHV genome (revised line 474).

6. The authors has focused the discussion on the significance of the role of alternation of the KSHV sequences in KS tumorigenesis and potential immune escape mechanisms by the virus. However, more emphasis should be placed on tumor DNA sequences instability as a result of rapid tumor cell growth and tumorigenesis, and may not have anything to do with viral pathogenesis and tumorigenesis. Moreover, it has been shown that KS tumor cell cultures without detectable KSHV sequences can be established. Thus, changes of KSHV sequences could occur after tumor establishments.

Response: We agree and addressed similar concerns made by Reviewers 1 and 2 (please see in particular response to major comment 1 of Reviewer #2).

7. The authors have suggested that since K8.1 can generate an effective immune response and thus alternations in K8.1 gene could provide an advantage for viral escape, pathogenesis and tumor growth. However, it has been shown that KS patients still mount a robust humoral immune response against KS, including against K8.1. Thus, it is unlikely that changes in K8.1 paly an important with KS tumorigenesis.

Response: We agree, suppressing K8.1 expression may better allow continued proliferation of the transformed host cell by avoiding the immune response. Please see response to minor comment 4 of Reviewer # 2, and current lines 557-559.

8. The result showing that in some cases more than one tumor lesion in the same patient harbors the same KSHV alternations is interesting. This suggests that KS lesions could be clonal origin due to metastases, but could also arise independently. Such points should be emphasized in the discussion.

Response: We contend that they are most likely to be clonally related, as the odds of independently acquiring the same mutation with the same breakpoint coordinates are infinitesimal. We highlight this finding more throughout the manuscript (e.g. new paragraph from line 560); please see also our response to minor comment 12 of Reviewer# 1.

Minor comments:

1. Table 2 title needs to be changed. It titled as gene copy in tumor DNA but the table showed oral KSHV sequences which are not from tumors.

Response: We modified the table title following the reviewer’s comment.
