# Supplementary material for: Intra-host changes in Kaposi sarcoma-associated herpesvirus genomes in Ugandan adults with Kaposi sarcoma
Source: PLoS Pathog. 2021 Jan 19;17(1):e1008594. doi: 10.1371/journal.ppat.1008594 (PMC7845968; doi:10.1371/journal.ppat.1008594)
Supplement: Attachment — Submitted filename: Reviewer_response2.docx [file ppat.1008594.s013.docx]

**Response to Reviews:**

**Part I - Summary**

Reviewer #1: The authors have addressed the reviewers' concerns.

Reviewer #3:

1. This reviewer agrees with the previous reviewers that the findings on the deletions, duplications and changes are most likely due to the result of genome instability of the tumor cells rather than the cause of tumorigenesis. Similar changes in multiple lesions can be the results of the metastases of tumor cells harboring the mutations in the KSHV genome. This was demonstrated by case U003 that only 3 out of 5 lesions have the mutations and the other two did not, and were similar to the saliva KSHV sequences. This suggests that KS can develop in the absence of changes in the KSHV genome, and changes have likely occurred after tumorigenesis, and thus KSHV genome changes may not have substantial influence on pathogenesis. Further discussing this possibility will be helpful.

*The reviewer misunderstood this data: As we reported, all 5 tumors tested of U003 had the K8.1-TR junction indicative of the U003-C genomic inversion, and only 3 of the 5 had intact K8.1 sequence as well (lines 579-581). Nevertheless, we further stressed the possibility that tumor genomic instability may have caused the KSHV genomic aberrations in the Abstract, Author Summary and Discussion.*

2. As one of the reviewer has pointed out “the justification for doing the work and for doing the work at the incredible level of sophistication was somewhat lacking ….” Further strengthening the rationale will be useful.

*We used these emerging technologies to both define viral genetic variation at the whole genome level and help resolve prior controversies in KSHV intra-host diversity that were unclear with the use of older less sophisticated sequencing techniques that introduce errors possibly greater than that which occurs in the viral population in vivo. We further stressed the value of our approach in the Abstract, Author Summary, Introduction and Discussion.*

3. Line 265, are the authors referring to Fig. 1B rather than Fig. 3B?

*It should be 1B. We corrected this error and thank the reviewer.*

**Part II – Major Issues: Key Experiments Required for Acceptance**

Reviewer #1: NA

Reviewer #3: (No Response)

**Part III – Minor Issues: Editorial and Data Presentation Modifications**

Reviewer #1: The conflict of interested statement should be expanded upon to explain that the authors company expressly markets the technology used in this manuscript.

The statement previously submitted is now revised with the addition of the phrase underlined below:

*I have read the journal's policy and the authors of this manuscript have the following competing interests: MWS is a founder and equity holder at TwinStrand Biosciences, Inc., which is commercializing Duplex Sequencing technology*

Reviewer #3: (No Response)
